# D-Limonene Exhibits Antiproliferative Activity Against Human Colorectal Adenocarcinoma (Caco-2) Cells via Regulation of Inflammatory and Apoptotic Pathways

**DOI:** 10.3390/cimb47050370

**Published:** 2025-05-18

**Authors:** Abdullah A. A. Alghamdi

**Affiliations:** Department of Biology, Faculty of Science, Al-Baha University, Al-Baha 65779, Saudi Arabia; aaa.alghamdi@bu.edu.sa

**Keywords:** D-limonene, colorectal cancer, GSH, TNF-alpha, Bax/Bcl2, p53, LDH, Ki67, MMP2, MMP9

## Abstract

Current therapies for colorectal cancer (CRC) are associated with significant side effects and limitations, driving the search for novel therapeutic approaches. This study investigated the antiproliferative potential of D-limonene, a natural compound, on human colorectal adenocarcinoma (Caco-2) cells and analyzed its underlying mechanisms. Caco-2 cells were treated with D-limonene or doxorubicin (DOX) for 24 h. Cell viability was assessed using the MTT assay, with D-limonene and DOX showing IC50 values of 18.6 and 6.4 µM, respectively. In comparison to controls, D-limonene treatment dramatically enhanced the formation of reactive oxygen species (ROS) and decreased cellular antioxidant capacity, as seen by concentration-dependent lower glutathione (GSH) levels. The substance also increased the levels of pro-apoptotic proteins (caspase-3, Bax), tumor suppressor p53, lactate dehydrogenase (LDH), and inflammatory indicators [tumor necrosis factor alpha (TNF-α) and interleukin-1 beta (IL-1β)]. Furthermore, in a concentration-dependent way, D-limonene therapy decreased the levels of matrix metalloproteinases (MMP2, MMP9), proliferation marker Ki67, and the anti-apoptotic protein Bcl-2. These results imply that the induction of oxidative stress, inflammation, and apoptotic pathways mediates D-limonene’s antiproliferative actions in colon cancer cells. Our findings show that D-limonene has therapeutic promise as a natural substitute for the treatment of colorectal cancer.

## 1. Introduction

Cancer represents the leading cause of mortality in the 21st century, characterized by uncontrolled proliferation and dissemination of abnormal cells worldwide. Globally, there were almost 9.5 million cancer-related deaths and 18.07 million new cancer diagnoses, according to the International Agency for Research on Cancer (IARC). With an annual mortality rate of 9.4%, colorectal malignancies (CRCs) are the third most common kind of cancer. Over 1.9 million new cases of colorectal cancer and 930,000 deaths were predicted for 2020 [1]. Among Gulf nations, Saudi Arabia demonstrates one of the highest age-standardized incidence rates (ASIRs) for both genders, according to ICAR, WHO, and GLOBOCON 2018 [2]. In addition to non-modifiable factors like colon polyps (25–30%), ulcerative colitis, Crohn’s disease, and hereditary syndromes (approximately 5% of cases), risk factors for colorectal cancer (CRC) include modifiable factors (60–65% of sporadic cases) like obesity, high consumption of red and processed meats, alcohol consumption, tobacco use, and inflammatory bowel disease [3]. Additional factors contributing to rising CRC incidence include westernization, altered dietary and lifestyle patterns, reduced physical activity, industrialization, and environmental factors [4]. While conventional cancer treatments encompass chemotherapy, radiation therapy, hormone therapy, and surgery, their associated resistance and adverse effects have necessitated the search for alternative therapeutic approaches [5]. Natural products remain a valuable source of potential chemotherapeutic agents with significant pharmacological importance [6].

**Figure 1:**
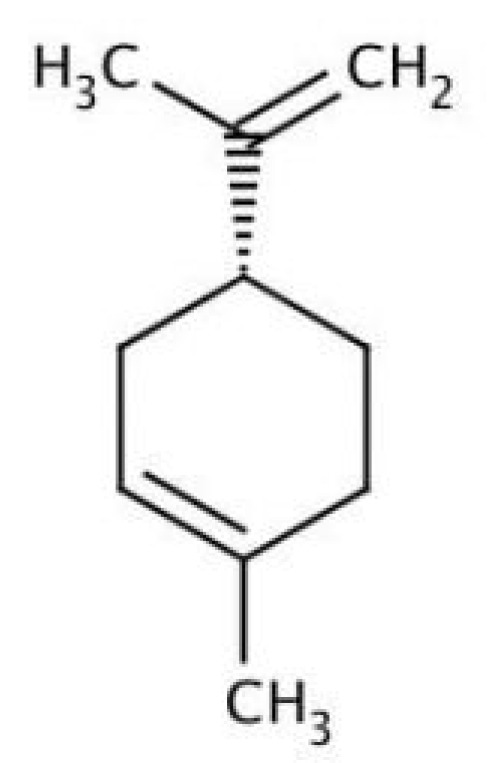
Chemical structure of D-limonene.

D-limonene, a monoterpene abundant in citrus fruit peels such as orange and lemon, exists in two isomeric forms (D and L). The more bioactive form, chemically known as 1-methyl-4-(1-methylethenyl) cyclohexene (Figure 1), is widely used as a flavoring agent and food additive in beverages, confectioneries, cosmetics, and personal care products due to its citrus fragrance [7]. D-limonene demonstrates various therapeutic properties, including antioxidant effects that reduce lipid peroxidation and free radical damage, making it effective against physical and psychological stress and stress-related hypertension [8,9]. Recent studies have also highlighted D-limonene’s potential role in managing pulmonary hypertension [10] and its chemotherapeutic effects against various cancers, including colon and gastric malignancies [7,11]. Kummer et al. [12] demonstrated D-limonene’s ability to reduce tumor necrosis factor alpha (TNF-a) levels and inhibit neutrophil and leukocyte chemotaxis in zymosan-induced peritonitis. Piccinelli et al. revealed D-limonene’s unexpected antihyperalgesic and antidepressant effects in rat models of neuropathic pain using the spared nerve injury (SNI) paradigm. In vitro studies examining D-limonene’s immunomodulatory properties showed suppression of TH1 and TH2 cytokine production by stimulated T cells [13].

Research has established D-limonene’s diverse therapeutic properties, including antiproliferative, antioxidant, anti-inflammatory, anti-apoptotic, and antidiabetic activities. It protects cells by scavenging reactive oxygen species and preventing mitochondrial dysfunction. Additional biological properties include hypolipidemic, immunomodulatory, hepatoprotective, dermatoprotective, and chemopreventive effects. Studies have also demonstrated D-limonene’s significant role in ameliorating gallstone formation and doxorubicin-induced nephrotoxicity [8,14,15,16].

This study aims to investigate D-limonene’s antiproliferative effects on colon cancer Caco-2 cells, specifically examining its influence on oxidative stress, inflammation, and apoptotic pathways.

## 2. Materials and Methods

### 2.1. D-Limonene Preparation

Sigma-Aldrich (St. Louis, MO, USA) was the supplier of D-limonene with a 97% purity level. Prior to experiments, Dulbecco’s modified Eagle’s medium (DMEM) (Sigma-Aldrich, St. Louis, MO, USA) was used to dilute the stock solution in order to reach the required final concentrations.

### 2.2. Caco-2 Cell Culture

Under standard circumstances, Caco-2 cells were cultivated in Dulbecco’s modified Eagle’s medium (DMEM), which was supplied by VACSERA. A total of 10% heat-inactivated fetal bovine serum (FBS), 4 mM glutamine, and antibiotics (100 IU/mL streptomycin and 100 IU/mL penicillin) were added to the medium. The cells were kept at 37 °C with a 5% CO_2_ environment in a humidified incubator. Every two to three days, sub-culturing was carried out [5]. Cell culture reagents were acquired from Lonza^®^.

### 2.3. Cell Viability Assay

The highest half inhibitory concentration (IC50) of D-limonene and doxorubicin on HEK-293, HCT-116, and Caco-2 cells was ascertained using the MTT test. A total of 200 μL of culture media was used to seed 1.5 × 10^4^ Caco-2 cancer cells per well in 96-well culture plates, and the cells were incubated for 24 h. Cells were then subjected to different concentrations of D-limonene and doxorubicin (0, 0.01, 0.1, 1.0, 10.0, 20.0, and 50.0 μg/mL) for 24 h at 37 °C in an environment with 5% CO_2_. The only negative control was DMEM. The medium was changed to 5 mg/mL of 3-(4,5-dimethylthiazol-2-yl)-2,5-diphenyl-2H-tetrazolium bromide (MTT) reagent (Life Technologies, Madrid, Spain) in full medium after adherent cells had been drug-treated for 24 h. The cells were then incubated for 4 h. The absorbance of the resulting purple formazan was measured at 570 nm using a microplate reader (17). Cell viability was calculated using the following formula: viability % = [(treated sample OD/untreated sample OD) × 100], with cell survival expressed as a percentage relative to the control. The 24 h treatment period was selected to evaluate the early mechanistic effects of D-limonene, including ROS generation, apoptosis induction, and inflammatory modulation, which are consistent with prior studies demonstrating rapid responses to this compound [17,18,19]. While the doubling time of Caco-2 cells is approximately 18–20 h, our focus was on acute cytotoxic and pro-apoptotic events rather than long-term proliferation inhibition. Future studies will incorporate extended exposure times to assess cumulative effects on cell viability and metastatic potential.

### 2.4. Reactive Oxygen Species Exploration

The accumulation of intracellular reactive oxygen species (ROS) in Caco-2 cells was assessed using the green, fluorescent probe 2,7-dichlorofluorescein diacetate (DCFH-DA). Cells were treated with doxorubicin at ½ IC50, D-limonene at ½ IC50, and D-limonene at 1/3 IC50 concentrations. Following 24 h of treatment, cells underwent two washes with Hanks’ balanced salt solution and were subsequently incubated with 10 μM DCFH-DA in Dulbecco’s modified Eagle’s medium (DMEM) for 30 min at 37 °C. After two washes with phosphate-buffered saline (PBS), the cells were analyzed. Reactive oxygen species (ROS) facilitate the conversion of DCFH-DA to dichlorofluorescein (DCF), a compound characterized by high fluorescence and impermeability to membranes. Fluorescence was detected utilizing a Nikon Eclipse fluorescence microscope (Nikon Corporation, Tokyo, Japan), with excitation and emission wavelengths set at 488 nm and 530 nm, respectively. The mean fluorescence intensity of DCF was quantified using a fluorescence plate reader [20].

### 2.5. Reduced Glutathione (GSH) Detection

Using Ellman’s method, the amount of glutathione (GSH) in Caco-2 cell lysates was determined [21]. Phosphate buffer and 5,5′-dithiobis-(2-nitrobenzoic acid) (DTNB, Sigma-Aldrich), commonly referred to as Ellman’s reagent, were mixed with cell extracts. At 412 nm, the resultant chromogenic product was quantified using spectrophotometry. Caco-2 cells were washed with phosphate-buffered saline (PBS) prior to analysis, and then they were homogenized in a 50 mM Tris-HCl solution at pH 7.4. The Lowry technique was used to measure the amount of protein present in the supernatants [22]. Nanomoles per milligram of protein was the unit of measurement for GSH levels.

### 2.6. Assessment of Pro-Inflammatory Markers

Following the manufacturer’s instructions (Abcam, Cambridge, UK), the enzyme-linked immunosorbent assay was used to determine the quantities of pro-inflammatory and immuno-regulator cytokines TNF-alpha and interleukin-1 beta (IL-1 β) in the supernatant (cat. no. ab285312 and ab214025).

### 2.7. Estimation of Apoptotic/Anti-Apoptotic Markers

Following the manufacturer’s instructions, the enzyme-linked immunosorbent assay (ELISA) kit (Abcam Company, Cambridge, UK) was used to detect the levels of B-cell lymphoma 2 (Bcl-2) (cat. no. ab272102), Bcl-2-associated X protein (Bax) (cat. no. ab199080), p53 (cat. no. ab171571), and caspase-3 (cat. no. NBP2-75,024). The ratio Bax/Bcl2 was then computed.

### 2.8. Gene Expression Analysis

Using quantitative real-time PCR, the expression levels of the apoptotic genes Bax, caspase-3, Bcl-2, and P53 were assessed in Caco-2 cells. TRIzol reagent (Qiagen, Germantown, MD, USA) was used to extract total RNA from Caco-2 cells in accordance with the manufacturer’s instructions. Nanodrop spectrophotometry was used to measure the amount of RNA present. The RevertAidTM H Minus Reverse Transcriptase kit (Fermentas, ThermoFisher Scientific Inc., Mississauga, ON, Canada) was then used to synthesize cDNA. Using a ViiATM 7 PCR system (Applied Biosystems, Foster City, CA, USA) and a SYBR green PCR kit (Qiagen, Hilden, Germany), gene expression was assessed. Every reaction was carried out in triplicate. GAPDH was used as the housekeeping gene for standardization when calculating relative gene expression using the 2^−ΔΔ^Ct technique. Table 1 lists the specific primer sequences that were employed for amplification.

### 2.9. Valuation of Lactate Dehydrogenase (LDH)

The lactate dehydrogenase (LDH) ELISA kit (cat. no. MBS269509) was used to detect LDH enzyme levels, following the manufacturer’s instructions (MyBioSource, Inc., San Diego, CA, USA).

### 2.10. Estimation of Cellular Proliferation Marker (Ki67)

The cellular proliferation marker (Ki67) was evaluated using the human ELISA kit (Abcam, Cambridge, UK) (cat. no. ab253221) following the manufacturer’s instructions.

### 2.11. Assessment of Matrix Metalloproteinases

Matrix metalloproteinase2 (MMP2) and matrix metalloproteinase9 (MMP9) levels were detected in the supernatant using ELISA kits following the manufacturer’s instructions (Abcam, Cambridge, UK) as the MMP2 ELISA kit (cat. no. ab100606) and human MMP9 ELISA kit (cat. no. ab246539), respectively.

### 2.12. Statistical Analysis

Version 17 of the Statistical Package for the Social Sciences (SPSS) program was used to statistically analyze the data. The mean ± standard deviation (SD) was used to represent the data. The one-way analysis of variance (ANOVA) test and the Tukey’s post hoc test were used to evaluate the statistical significance of the differences between the groups. A *p*-value of less than 0.05 was deemed significant.

## 3. Results

### 3.1. Cytotoxic Activity of D-Limonene

**Figure 2:**
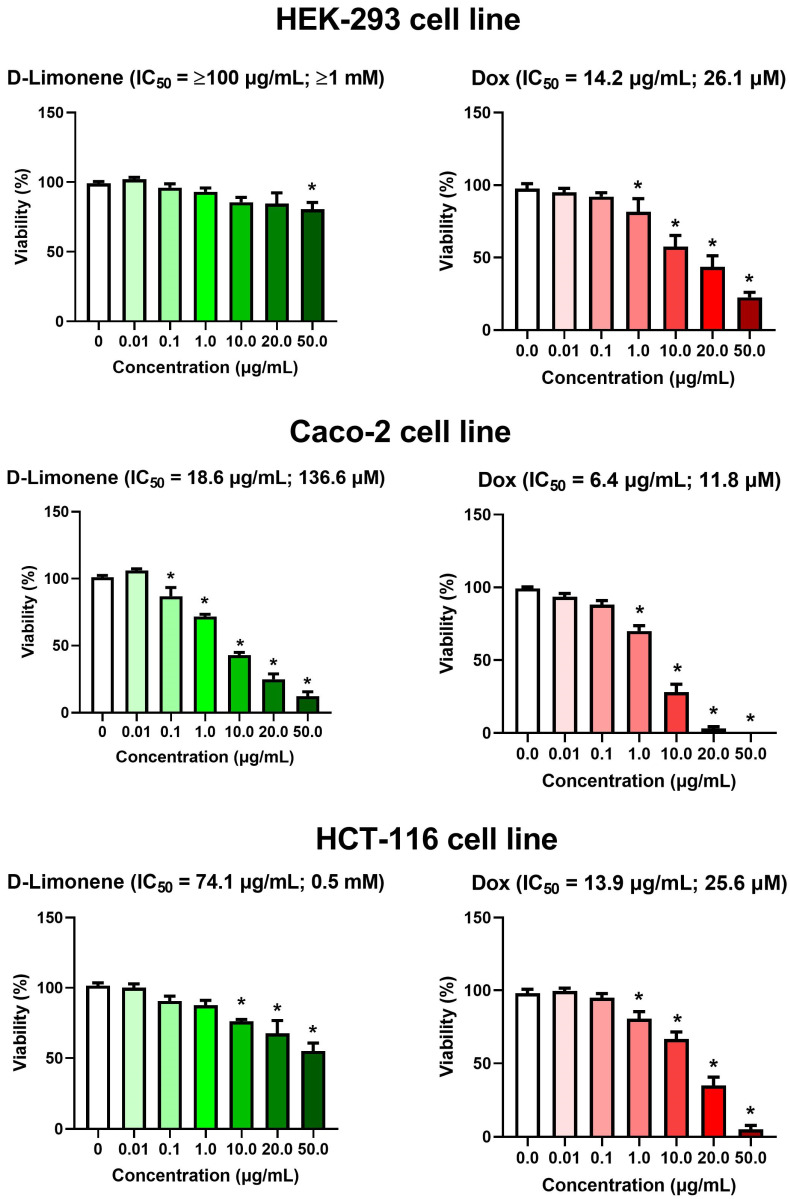
Effects of different concentrations of Dox and D-limonene on the viability of the HEK-293, Caco-2, and HCT-116 cell lines using an MTT assay. Values represent the mean ± standard deviation (SD) of the two experiments. * Represent the significant difference between different concentrations at *p* ˂ 0.05.

The cytotoxic potential of D-limonene against multiple cell lines was evaluated alongside doxorubicin (Dox), an anthracycline antibiotic used as a positive control, for 24 h. As shown in the Figure 2, D-limonene exhibited varying degrees of cytotoxicity across the tested cell lines, with IC50 values of ≥100 μg/mL (≥1 mM) for HEK-293, 18.6 μg/mL (136.6 μM) for Caco-2, and 74.1 μg/mL (0.5 mM) for HCT-116 cells. In comparison, doxorubicin demonstrated consistently higher cytotoxicity with IC50 values of 14.2 μg/mL (26.1 μM) for HEK-293, 6.4 μg/mL (11.8 μM) for Caco-2, and 13.9 μg/mL (25.6 μM) for HCT-116 cells. Notably, the Caco-2 cell line showed the highest sensitivity to both compounds, with significant reductions in viability observed at concentrations as low as 0.1 μg/mL for D-limonene and 1.0 μg/mL for doxorubicin. In contrast, HEK-293 cells were most resistant to D-limonene treatment, maintaining over 80% viability even at 20.0 μg/mL, with significant reduction only observed at 50.0 μg/mL. The HCT-116 cell line showed moderate sensitivity to both compounds, with significant decreases in viability at concentrations of 10.0 μg/mL and above.

### 3.2. Effects of D-Limonene on Oxidant–Antioxidant Balance

**Figure 3:**
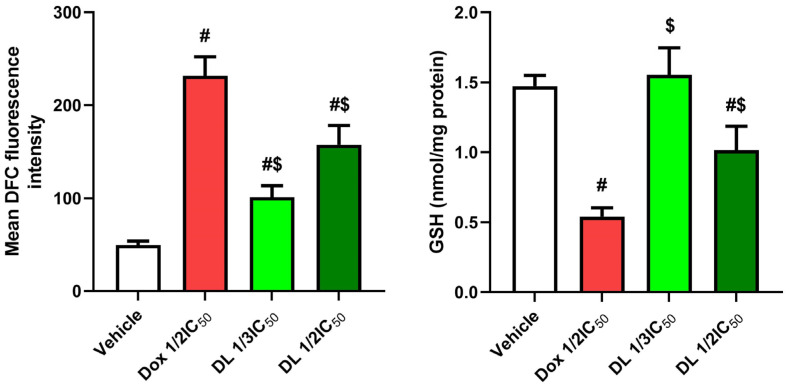
Effects of Dox ½ IC50, D-limonene ½ IC50, and D-limonene 1/3 IC50 treatment on ROS and GSH in the Caco-2 cell line. Values represent the mean ± standard deviation (SD) of the experiments. #: significant with respect to the control (*p* ˂ 0.05). $: significant with respect to Dox ½ IC50 (*p* ˂ 0.05).

D-limonene at ½ IC50 induced a considerable raise in ROS levels (*p* < 0.05) compared to D-limonene 1/3 IC50 and control groups. This was accompanied by significant depletion of cellular antioxidant capacity, as evidenced by decreased GSH content (*p* < 0.05) in a concentration-dependent manner. DOX ½ IC50 showed reduced GSH levels compared to controls, while D-limonene ½ IC50 exhibited significantly lower GSH levels compared to D-limonene 1/3 IC50-treated cells (Figure 3).

### 3.3. Impact of D-Limonene on Pro-Inflammatory Biomarkers

**Figure 4:**
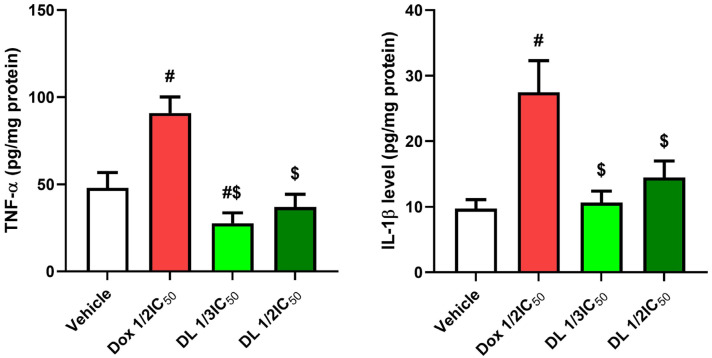
Effects of Dox ½ IC50, D-limonene ½ IC50, and D-limonene 1/3 IC50 treatment on TNF-alpha and IL-1 beta in the Caco-2 cell line. Values represent the mean ± standard deviation (SD) of the experiments. #: significant with respect to the control (*p* ˂ 0.05). $: significant with respect to Dox ½ IC50 (*p* ˂ 0.05).

Treatment with DOX ½ IC50 significantly elevated inflammatory markers (TNF-α and IL-1β) compared to control cells (*p* < 0.05). D-limonene 1/3 IC50 treatment resulted in substantially diminished TNF-α and IL-1β levels (*p* < 0.05) compared to DOX ½ IC50-treated cells, with levels approaching those of control cells (Figure 4).

### 3.4. D-Limonene’s Effect on Apoptotic Markers

**Figure 5:**
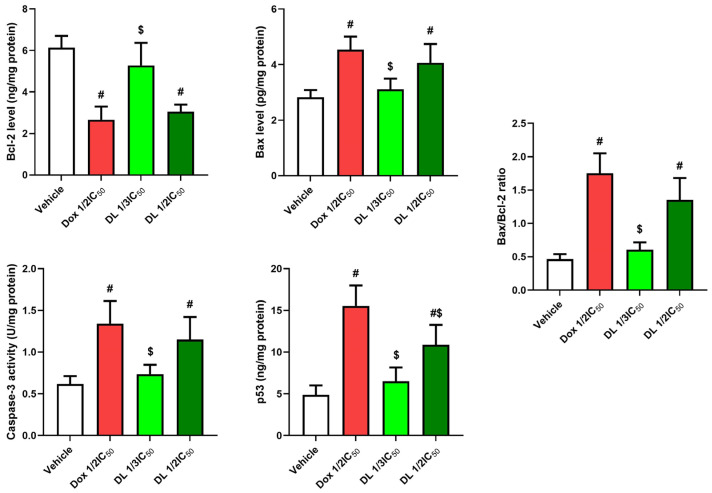
Effects of Dox ½ IC50, D-limonene ½ IC50, and D-limonene 1/3 IC50 treatment on levels of Bcl-2, Bax, Cas-3, p53, and Bax/Bcl-2 ratio expressions in the Caco-2 cell line. Values represent the mean ± standard deviation (SD) of the experiments. #: significant with respect to the control (*p* ˂ 0.05). $: significant with respect to Dox1/2 IC50 (*p* ˂ 0.05).

Treatment with D-limonene (½ IC50 and 1/3 IC50) and doxorubicin (Dox ½ IC50) significantly altered the expression of apoptosis-related genes. The anti-apoptotic marker, Bcl-2, showed significant reduction in all treatment groups, with doxorubicin showing the most pronounced decrease compared to vehicle control. D-limonene treatment at both concentrations resulted in moderate but significant decreases in Bcl-2 expression (Figure 5).

**Figure 6:**
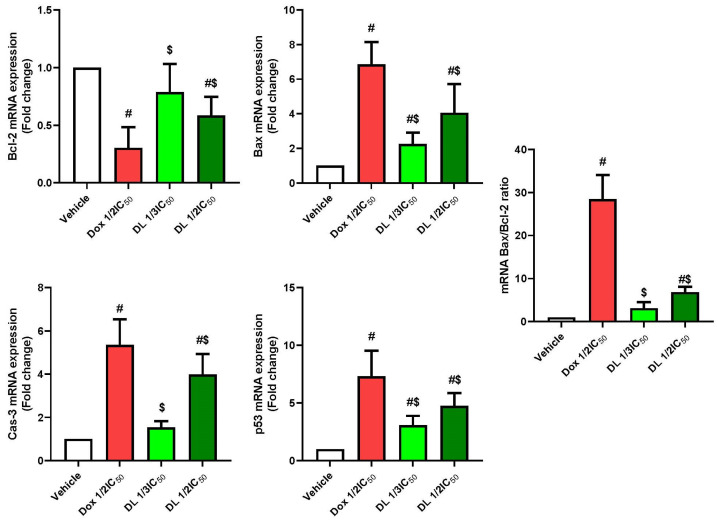
Effects of Dox ½ IC50, D-limonene ½ IC50, and D-limonene 1/3 IC50 treatment on mRNA expression of Bcl-2, Bax, Cas-3, p53, and Bax/Bcl-2 ratio expressions in the Caco-2 cell line. Values represent the mean ± standard deviation (SD) of the experiments. #: significant with respect to the control (*p* ˂ 0.05). $: significant with respect to Dox1/2 IC50 (*p* ˂ 0.05).

Pro-apoptotic markers demonstrated significant upregulation. Bax mRNA expression markedly increased in all treatment groups, with doxorubicin showing approximately a 7-fold increase, while D-limonene demonstrated concentration-dependent increases at ½ IC50 (4-fold) and 1/3 IC50 (2-fold) compared to untreated cells. Similarly, caspase-3 mRNA expression was significantly elevated, showing a 5-fold increase with doxorubicin, 4-fold with D-limonene ½ IC50, and 1.5-fold with D-limonene 1/3 IC50. The tumor suppressor p53 mRNA levels also significantly increased across all treatments, with doxorubicin inducing a 7-fold increase, while D-limonene showed concentration-dependent increases at ½ IC50 (5-fold) and 1/3 IC50 (3-fold). These molecular changes were reflected in the Bax/Bcl-2 ratio, a key indicator of apoptotic potential, which was dramatically elevated in the doxorubicin group (28-fold) and showed concentration-dependent increases in D-limonene-treated groups (Figure 5 and Figure 6).

### 3.5. Effects on LDH, Matrix Metalloproteinases, and Ki67

**Figure 7:**
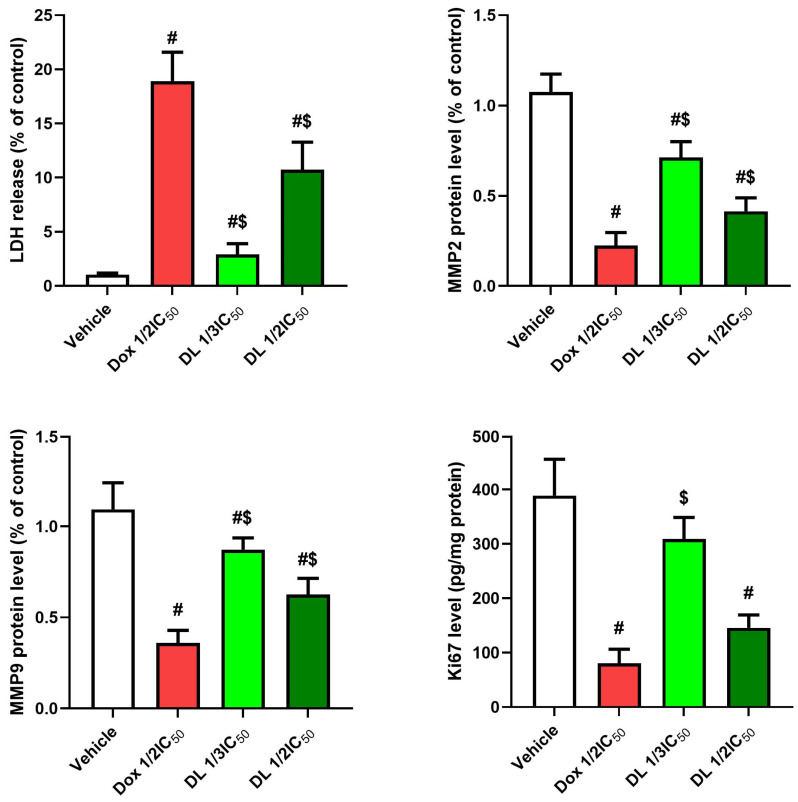
Effects of Dox ½ IC50, D-limonene ½ IC50, and D-limonene 1/3 IC50 treatment on LDH, MMP2, MMP9, and Ki67 in the Caco-2 cell line. Values represent the mean ± standard deviation (SD) of the experiments. #: significant with respect to the control (*p* ˂ 0.05). $: significant with respect to Dox ½ IC50 (*p* ˂ 0.05).

Lactate dehydrogenase (LDH) levels significantly increased (*p* < 0.05) in both D-limonene treatment groups compared to untreated controls, showing a concentration-dependent response. Conversely, matrix metalloproteinases (MMP2, MMP9) and the cellular proliferation marker Ki67 showed significant decreases (*p* < 0.05) in both DOX ½ IC50 and D-limonene-treated groups compared to untreated controls (Figure 7).

## 4. Discussion

Collective evidence from our research demonstrates that D-limonene inhibits intestinal cancer cell proliferation in culture. We further explored the underlying mechanism, showing that D-limonene triggers apoptosis through the induction of free radical generation. Currently, worldwide data indicate that colorectal cancer, along with other neoplastic diseases, represents the greatest health threat to humanity, affecting millions of individuals. Recent studies have investigated various colorectal cancer treatments; however, these approaches have not demonstrated sufficient efficacy to warrant pharmaceutical development for patient use [4]. Plant-derived compounds like D-limonene represent a valuable source of novel chemotherapeutic agents due to their structural diversity and unique mechanisms of action [23,24]. While some natural products show differential toxicity between malignant and non-malignant cells, as observed in the current study where D-limonene exhibited selective cytotoxicity against Caco-2 cells (IC50 = 136.6 μM) compared to its effects on HEK-293 (1000 μM) and HCT-116 (500 μM), this selectivity must be empirically validated for each compound. The pharmacological potential of such agents should be evaluated based on their specific therapeutic indices rather than assumptions about their natural origin [8,25].

In this study, we utilized the Caco-2 cell line as a colorectal cancer model to identify and evaluate D-limonene’s anti-proliferative effects. Our findings revealed significant differences between D-limonene treatment and negative controls, demonstrating its potency against cancer cells. Previous in vivo and in vitro studies have linked D-limonene to apoptosis induction [26,27,28]. Yu and colleagues [26] reported enhanced Bax expression in lung cancer cells following D-limonene treatment. Our research similarly showed increased levels of p53, Bax, and caspase-3 during D-limonene treatment, while Bcl-2 levels decreased, suggesting the mitochondria-mediated intrinsic death pathway plays a crucial role in D-limonene-induced Caco-2 cell death, consistent with its role in other malignancies.

Phase I clinical trials have shown D-limonene to be well-tolerated with consistent therapeutic activity in advanced cancer patients [29]. D-limonene combined with docetaxel enhanced hormone-resistant prostate cancer cell sensitivity without affecting healthy prostate epithelial cells [30]. Shah et al. [31] suggested that modification of proteins involved in the mitochondrial apoptotic pathway may explain the overall beneficial effect. Apoptosis, or programmed cell death, is known to participate in numerous biological processes. Most antiproliferative agents demonstrate the ability to induce apoptosis in tumor cells both in vivo and in vitro, correlating with their tumor growth inhibition capacity. Caspases are widely recognized as essential apoptosis mediators, with effector caspases like caspase-3 triggering PARP cleavage and ultimately leading to apoptosis [27]. Recent research indicates D-limonene may induce cellular death in certain cancers [32]. Our study demonstrated that D-limonene-induced apoptosis involves caspase activation. While D-limonene activated cleaved caspase-8, caspase-3, and PARP in LS174T cells, caspase-8 involvement appears unlikely [27], suggesting apoptosis occurred via a mitochondria-mediated pathway.

Treated Caco-2 cells showed decreased or unchanged pro-inflammatory cytokine levels compared to untreated cells, indicating D-limonene therapy inhibited pro-inflammatory cytokine production. The tumor microenvironment significantly influences breast cancer development [33]. Pro-inflammatory cytokines IL-1β and TNF-α serve as key inflammatory mediators and have been linked to cancer development in numerous studies [34,35]. These cytokines cooperatively form an interconnected network potentially affecting cancer cell growth [36].

Through a transglutaminase-2-dependent mechanism, IL-1β increases the aggressiveness of cancer cells in the tumor microenvironment by inducing the production of IL-6. In three-dimensional culture, anti-IL-1β antibody therapy decreased the invasiveness and survival of breast cancer cells [37]. In vivo, MCF-7 breast cancer cell proliferation and bone metastases development were decreased by IL-1β inhibition. Decreased IL-1β activity alters the tumor microenvironment, increasing cancer cell necrosis and reducing breast cancer angiogenesis and proliferation [38]. TNF-α demonstrates both growth inhibition and apoptosis induction in Caco-2 cells [39,40]. Previous research showed p53 contribution to TNF-α’s cytotoxic effects, with p53 function loss resulting in cancer cell resistance to TNF-induced death [41]. Wolczyk et al. reported that TNF-induced MAPK/ERK pathway activation facilitates cancer cell motility [42]. Our findings support previous research on pro-inflammatory cytokine expression. We found D-limonene significantly reduced IL-1β and TNF-α protein levels in Caco-2 cells, particularly at low IC50 concentrations. The TNF-α expression data align with studies showing moderate Caco-2 cell response to TNF-α [43], potentially due to variations in TNF receptor expression, Bcl-2 family protein expression, and protease activation.

Various cancers show notably increased MMP gene expression and activity, particularly MMP9. Elevated MMP9 levels positively correlate with poor cancer prognosis, suggesting its potential as a marker for breast, colorectal, ovarian, and non-small cell lung cancer. Consequently, MMP9 inhibition represents a promising antiproliferative strategy [44]. Studies show MMP9 can compromise tight junction integrity in Caco-2 cells, affecting cell polarity and epithelial barrier function [45]. CRC patient mortality largely results from metastasis and distant tumor expansion. MMP-2 and MMP-9 correlate with Caco-2 cell invasion and metastasis [5]. Our results showed D-limonene significantly reduced MMP-2 and MMP-9 protein levels in Caco-2 cells, suggesting D-limonene may prevent Caco-2 cell migration and invasion through MMP downregulation.

Many antiproliferative drugs induce apoptotic cell death by elevating oxidative stress to levels that compromise cell viability and disrupt the ROS–antioxidant balance in cancer cells. Baseline ROS levels activate pathways for cell growth, survival, differentiation, death, motility, immune responses, and stress response. However, increased ROS leads to cell death by affecting viability. Our data show GSH content increased at D-limonene 1/3 IC50 and decreased at 1/2 IC50. D-limonene appears to activate antioxidant defense systems in Caco-2 cells through enhanced cellular oxidative stress. Despite antioxidant protection, Caco-2 cells underwent apoptosis, suggesting D-limonene-induced ROS levels exceeded antioxidant system capacity. Higher D-limonene concentrations generate ROS levels that trigger apoptotic pathways, preventing Caco-2 cancer cell survival, proliferation, and viability. Many chemotherapy drugs are known to induce oxidative stress in cancer cells [24], with low ROS levels maintaining cancer stem cell characteristics and promoting carcinogenesis [46].

## 5. Conclusions

This study demonstrates that D-limonene exhibits concentration-dependent antiproliferative activity against colorectal cancer cells. The compound’s therapeutic effects are mediated through multiple mechanisms, including modulation of oxidative stress, induction of apoptosis, and regulation of inflammatory responses. These findings suggest that D-limonene may serve as a promising adjuvant therapeutic agent to enhance the efficacy of conventional cancer treatments. Future clinical studies are warranted to evaluate its potential role in combination therapy for cancer patients. However, future studies are required to extend time points to assess cumulative effects on cell proliferation.

## Figures and Tables

**Table 1:** List of examined genes.

Name	Accession No.	Sense (5′–3′)	Antisense (5′–3′)
*P53*	NM_000546.6	GACACGCTTCCCTGGATTGG	GCTCGACGCTAGGATCTGAC
*Bcl2*	NM_000633.3	AAAAATACAACATCACAGAGGAAGT	GTTTCCCCCTTGGCATGAGA
*Casp3*	NM_001354777.2	GCGGATGGGTGCTATTGTGA	ACACAGCCACAGGTATGAGC
*Bax*	NM_001291428.2	ATGGACGGGTCCGGGG	GGAAAAAGACCTCTCGGGGG

## Data Availability

The original contributions presented in this study are included in this article. Further inquiries can be directed to the corresponding author.
